# National survey of clinical practices and perspectives in the management of ischaemic stroke patients with minor neurological deficits

**DOI:** 10.1007/s13760-025-02942-5

**Published:** 2026-01-07

**Authors:** Robert Croese, Juliëtte Santing, Korné Jellema, Heleen den Hertog

**Affiliations:** 1 Department of Neurology, Haaglanden Medical Centre, The Hague, The Netherlands; 2 Department of Neurology, Amsterdam University Medical Center, Amsterdam, The Netherlands; 3 Department of Neurology, Erasmus Medical Centre, Rotterdam, The Netherlands

**Keywords:** National survey, Acute ischaemic stroke management, Emergency department discharge, Clinical practice variation

## Abstract

**Background:**

Patients with ischaemic stroke are increasingly encountered in neurological practice. Current guidelines recommend hospital admission for monitoring and coordination of appropriate aftercare, regardless of stroke severity. However, the clinical necessity of admitting all such patients remains uncertain. Some neurologists argue that selected low-risk patients can be discharged directly from the emergency department (ED). Given the ongoing debate and lack of high-quality evidence, we aimed to explore current practices and attitudes toward the management of patients with ischaemic stroke with minor neurological deficits in the Netherlands.

**Methods:**

A national online survey was conducted to explore current variations in clinical practice regarding admission strategies for patients with ischaemic stroke. The survey included five clinical cases and was distributed to neurologists and neurology trainees. We assessed which patient and stroke characteristics influenced the decision to admit a patient.

**Results:**

A total of 107 respondents completed the survey, comprising 72 neurologists (67%) and 36 neurology trainees (33%). Admission rates for ischaemic stroke with minor neurological deficits varied considerably across the five clinical vignettes, ranging from 21% to 89%. Willingness to randomise patients into a trial evaluating admission versus ED discharge was high, with rates between 77% and 94% across cases. The majority of respondents (89%) considered a risk threshold of up to 5% for secondary deterioration acceptable to justify direct discharge from the ED. Of these, 44% would accept a risk of 1–2%, while 45% would accept a risk of 3–5%. Only 10% indicated that all ischaemic stroke patients should be admitted regardless of risk.

**Conclusion:**

Current guideline-driven practice in the Netherlands leads to hospital admission for nearly all patients with ischaemic stroke. However, our survey reveals substantial heterogeneity among neurologists and trainees regarding the willingness to consider direct ED discharge under specific conditions. If certain criteria are met, a majority would opt for ED discharge, with 89% accepting a risk of up to 5% for early neurological deterioration. These findings underscore the need for evidence-based admission strategies, potentially enabling safe ED discharge for selected patients and more efficient use of hospital resources.

## Introduction

Ischaemic stroke has become increasingly common in emergency departments (EDs) across Western countries, including the Netherlands [1]. Between 1990 and 2019, the absolute number of incident ischaemic stroke cases rose by 70%, while the total number of individuals living with the sequelae of stroke increased by 85%. This rise is largely due to population growth and ageing. In high-income countries, approximately 40 new ischaemic stroke cases per 100,000 inhabitants are diagnosed annually [2]. Despite this increase in absolute numbers, age-adjusted incidence and mortality rates have declined during the same period, reflecting the positive impact of advances in primary and secondary prevention, as well as acute stroke therapies [1, 2]. 

Current Dutch guidelines recommend that all patients with acute ischaemic stroke should be admitted to hospital, irrespective of severity, to enable continuous monitoring, diagnostic evaluation, rehabilitation, management of complications, and nursing care [3, 4]. Both the American Stroke Association (ASA) and the European Stroke Organisation (ESO) similarly advocate for the admission of all patients with acute ischaemic stroke [5, 6]. However, there is ongoing debate about the necessity of inpatient management for every patient, particularly those with minor neurological deficits.

The Dutch healthcare system is characterised by a well-organised stroke care pathway, yet despite this, considerable variation in clinical practice persists regarding the management of acute ischaemic stroke with minor neurological deficits [3]. Inpatient management may not always be required, especially for patients who do not need acute reperfusion therapies or inpatient rehabilitation. Emerging clinical evidence suggests that certain subgroups, such as those with minor neurological deficits, rapid neurological improvement, or no significant acute findings on neuroimaging, may be safely discharged directly from the ED following a period of observation [7, 8]. Furthermore, hospital admission carries inherent risks, including exposure to nosocomial infections and iatrogenic complications [9]. Selective discharge of carefully chosen patients with ischaemic stroke directly from the ED could therefore offer several benefits, including reduced healthcare costs, decreased clinical workload, and improved patient safety and satisfaction by mitigating these risks [7]. However, there are important considerations to address. Secondary complications could arise after discharge, such as symptomatic haemorrhagic transformation or recurrent ischaemic events [10, 11]. Direct discharge may also limit access to acute rehabilitation services and reduce opportunities for comprehensive patient education regarding secondary stroke prevention strategies [1]. 

This observed variation in clinical practice highlights the heterogeneity in both clinical practice and attitudes among neurologists and neurology trainees in the Netherlands concerning the management of patients with acute ischaemic stroke and minor neurological deficits. To address this, we conducted a national survey to systematically explore the factors influencing Dutch neurologists’ and trainees’ decision-making regarding direct ED discharge for acute ischaemic stroke patients with minor neurological deficits. This study seeks to identify the key patient and stroke characteristics that guide treatment decisions and to assess the extent to which current practice aligns with national guidelines. The findings will provide valuable insights into the complexities of clinical decision-making in this context and support the development of more standardised, evidence-based protocols.

## Methods

We conducted a national survey targeting neurologists and neurology trainees using the online platform Survio (http://www.survio.com/**)**, selected for its user-friendliness and ability to reach a wide audience of medical professionals across the Netherlands. Our objective was to present clinical cases reflecting common dilemmas in the acute management of ischaemic stroke, with a specific focus on the decision to admit or discharge.

The survey comprised three sections. The first section collected demographic and professional information about respondents. Demographic and professional characteristics of the respondents are summarized in Table 1. The second section presented five clinical cases of minor ischaemic stroke patients reflecting variability in symptom duration, neuroimaging, age, and National Institutes of Health Stroke Scale (NIHSS) score (see Table 2). Respondents were asked whether admission was necessary, which predefined factors influenced their decision, and their willingness to include patients in a clinical study. The primary endpoint was the admission decision per vignette. Secondary endpoints included willingness to randomise and selection of influencing factors from a predefined list (symptom progression, neuroimaging, ability to manage independently at home, blood pressure, social circumstances; see Table 3), with an option for free-text additional input. The third section asked respondents to indicate the maximum acceptable risk of secondary deterioration justifying direct ED discharge, with answer options of 1–2%, 3–5%, 6–10%, or ‘all patients should be admitted’. The survey instrument was developed jointly by vascular neurologists and neurology trainees to ensure clinical relevance and clarity. Sources of bias in case selection and phrasing were discussed in the team and with independent colleagues. No formal validation or pilot testing was performed. The full survey is provided in Appendix 1.

**Table 1:** Respondent characteristics

Responder’s characteristics	Trainees (*n* = 36)	Neurologists (*n* = 72)
Male, no. (%) Age group in years, no. (%) 20–29 30–39 40–49 50–59 60–70 Years since qualifying as a specialist, no. (%) 0–5 6–10 11–20 21–30 > 30 Department, no. (%) Academic hospital Major teaching hospital General hospital Specialist hospital	9 (25.0) 7 (19.4) 28 (77.8) 1 (2.8) 0 (0) 0 (0) - 11 (30.6) 25 (69.4) 0 (0) 0 (0)	31 (41.7) 0 (0) 18 (25.0) 31 (43.1) 16 (22.2) 7 (9.7) 18 (25.0) 15 (20.8) 26 (36.1) 12 (16.7) 1 (1.4) 11 (14.3) 46 (59.7) 20 (26.0) 0 (0)

**Table 2:** Questions, possible answers, and responses (proportions) with regard to the clinical cases

Questions	Possible answers	Answers (%)				
		Case 1	Case 2	Case 3	Case 4	Case 5
Would you admit this patient?	Yes No Other	68(63,6) 30(28,0) 9(8,4)	48(49,5) 42(43,3) 7(7,2)	34(34,3) 63(63,3) 2(2,0)	21(19,6) 81(75,7) 5(4,7)	89(83,2) 15(14,0) 3(2,8)
Would you be willing to leave this decision (whether or not to admit) open to randomisation in a study?	Yes No Other	83(77,6) 20(18,7) 4(3,7)	77(77,0) 30(28,0) 0(0)	94(80,3) 23(19,7) 0(0)	90(84,1) 16(15,0) 1(0,9)	37(35,2) 68(64,8) 1(1,0)

**Table 3:** Factors influencing admission decisions across clinical cases, with numbers and percentages of respondents indicating each factor’s influence on the decision to admit or not

Factor (Yes/No)	Case 1	Case 2	Case 3	Case 4	Case 5
Age	36(34%)/70 (66%)	29 (27%)/77 (73%)	42 (39%)/64 (60%)	23 (21%)/83 (78%)	18 (17%)/87 (81%)
Symptom duration	83 (78%)/23 (22%)	79 (74%)/27 (25%)	73 (68%)/33 (31%)	75 (70%)/31 (29%)	62 (58%)/44 (42%)
Symptom improvement	80 (75%)/26 (24%)	68 (64%)/38 (36%)	88 (82%)/10 (9%)	90 (84%)/16 (15%)	59 (55%)/47 (44%)
Social circumstances	78 (73%)/36 (33%)	45 (42%)/61 (57%)	65 (61%)/41 (38%)	39 (36%)/67 (62%)	32 (30%)/74 (69%)
Blood pressure	55 (51%)/51 (47%)	65 (61%)/41 (38%)	55 (51%)/51 (47%)	57 (53%)/49 (46%)	47 (44%)/60 (56%)
CT abnormalities	56 (52%)/50 (47%)	48 (45%)/56 (52%)	50 (47%)/56 (52%)	59 (55%)/47 (43%)	95 (89%)/11 (10%)

The survey was open from July to September 2024. It was distributed to neurology trainees (*n* = 337) and neurologists (*n* = 1,246) via the Netherlands Society of Neurology newsletter and through emails to members of the Neurovascular Workgroup (*n* = 151) and teaching hospitals (*n* = 15). To encourage participation and enhance representativeness, reminders were sent after two weeks and the importance of including diverse perspectives was emphasised. All responses were included in the analysis. The response rate was relatively low (approximately 7% of invited neurologists and trainees). To evaluate potential non-response bias, demographic characteristics of respondents were compared against available national data on Dutch neurologists and trainees, revealing no significant differences. The survey consisted exclusively of closed-ended questions, all collected data were quantitative and analysed using descriptive and comparative statistical methods.

Descriptive statistics were applied where appropriate. Statistical comparisons focusing on work experience and practice variation were conducted using the χ² test, Fisher’s exact test, and Fisher-Freeman-Halton exact test, as applicable. Analyses were performed using SPSS 28.0 (IBM, Chicago, IL, USA), with p-values < 0.05 considered significant. The minimal acceptable statistical accuracy was set at 15% with a 95% confidence interval, balancing the need for precision with feasibility given the timeframe and resources. Accuracy was calculated based on population size and response rates among neurologists and trainees; higher response rates improved accuracy, while larger populations required fewer responses to achieve similar accuracy compared to smaller populations.

## Results

### Respondents’ characteristics

A total of 107 respondents completed the survey on ischaemic stroke management, with 106 answering all questions. Of these, 72 (67%) were neurologists and 36 (33%) were neurology trainees. This distribution was intended to provide a representative overview of current clinical practices and perspectives on stroke management in the Netherlands. The baseline characteristics of the survey participants are presented in Table 1.

## Admission decisions and willingness to randomize

**Fig. 1:**
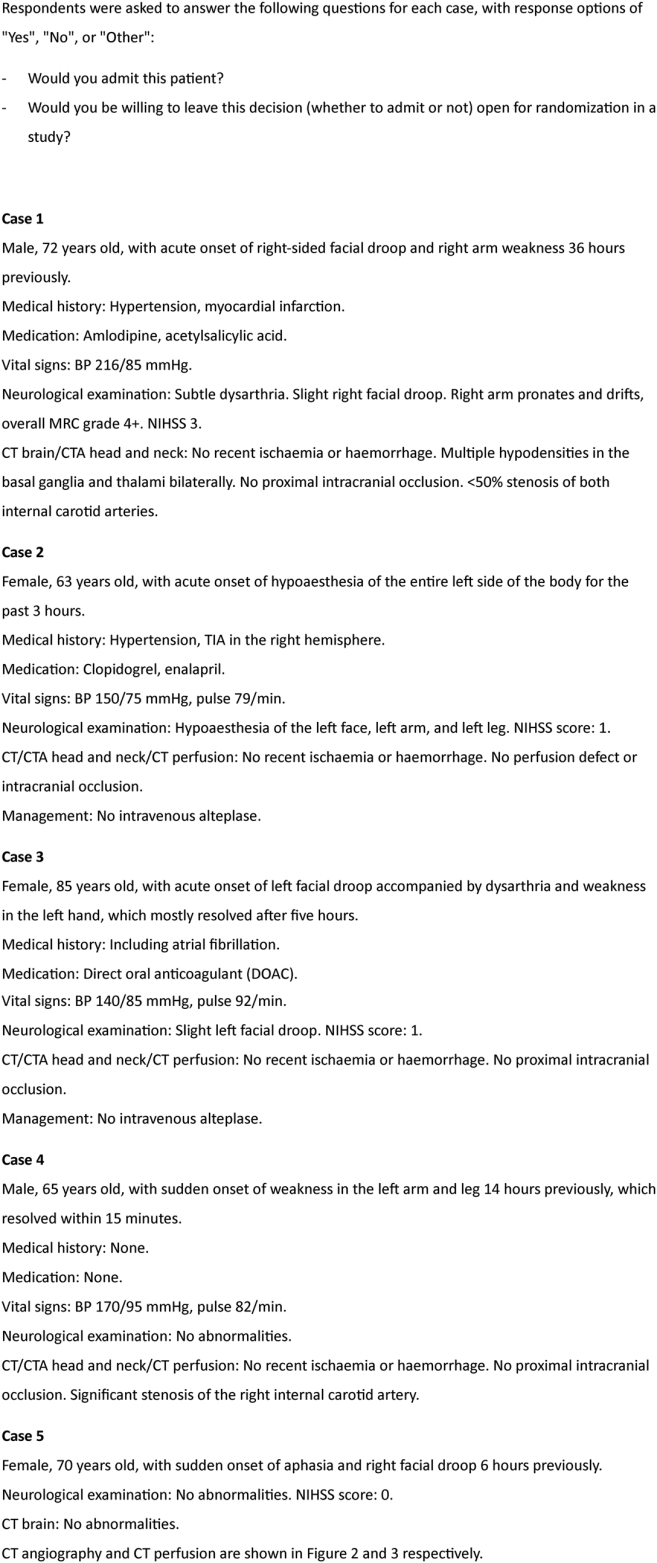
The five case vignettes and the accompanying CT scans. The clinical cases included: 1. A 72-year-old male with an NIHSS score of 3, presenting 36 hours post-symptom onset. 2. A 63-year-old female with an NIHSS score of 1, presenting 3 hours post-symptom onset. 3. An 85-year-old female with an NIHSS score of 1, presenting 5 hours post-symptom onset. 4. A 65-year-old patient with transient symptoms resolving within 15 minutes, presenting 14 hours post-onset. 5. A 70-year-old patient with an NIHSS score of 0, presenting 6 hours post-onset with no perfusion deficits on imaging (*overview questionnaire in appendix)

**Fig. 2:**
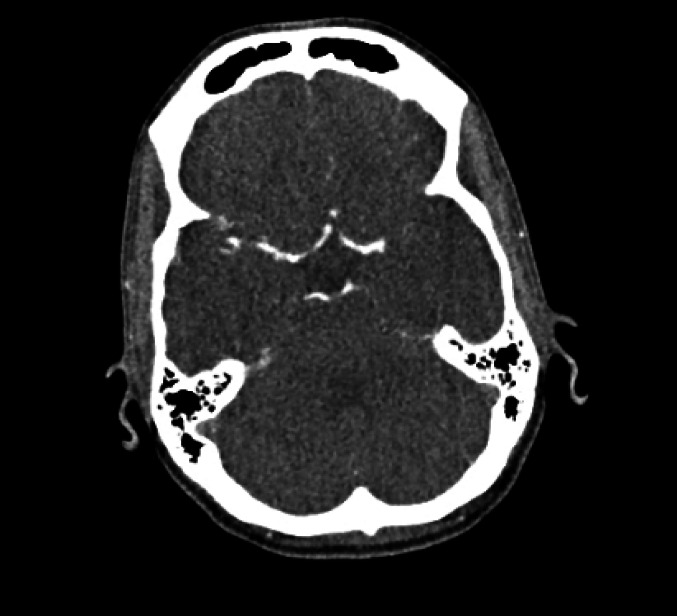
Axial CT angiography

**Fig. 3:**
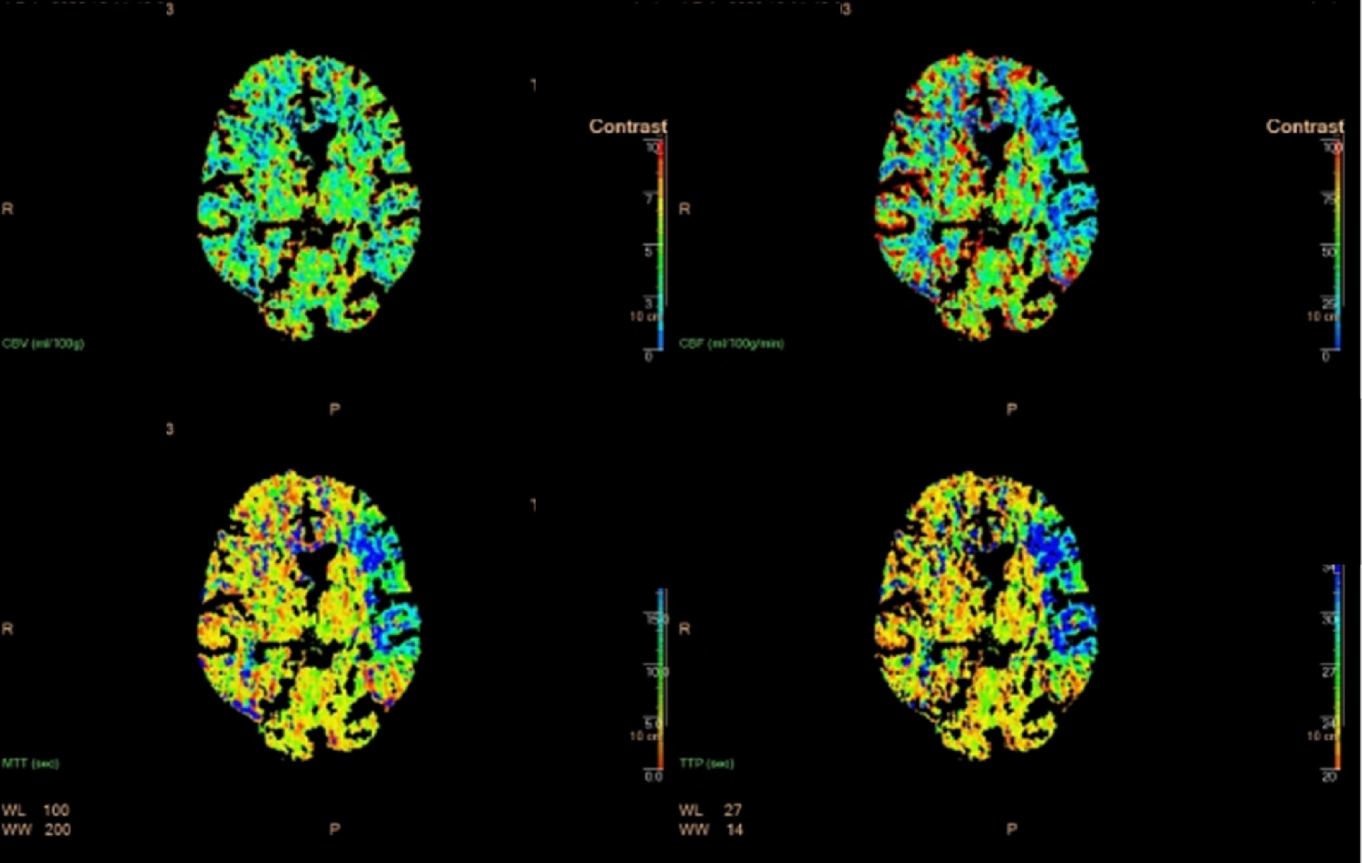
CT perfusion scan demonstrating, starting in the top left and proceeding clockwise: cerebral blood volume (CBV), cerebral blood flow (CBF), time to peak (TTP), and mean transit time (MTT)

Admission rates varied substantially across the five clinical vignettes, reflecting considerable heterogeneity in clinical decision-making (Table 2; Fig. 1). The lowest admission rate was observed in Case 4 (21%), featuring a patient with transient symptoms resolving within minutes. In contrast, Case 5, representing a patient with aphasia but no residual deficits, had the highest admission rate of 89%. Intermediate admission rates were reported for Cases 1, 2, and 3 at 68%, 50%, and 60%, respectively. Across cases, symptom duration and symptom improvement were the most frequently cited determinants, with social circumstances playing a considerable role. Blood pressure, neuroimaging abnormalities, and patient age showed variable influence depending on the vignette. An overview of the factors influencing admission decisions is provided in Table 3. Despite these variations, willingness to randomise patients into a trial evaluating admission versus ED discharge was consistently high, ranging from 77% to 94% across cases. For each case, key findings are detailed below Figs. 2 and 3.

## Case-specific findings

### Case 1

This case described a 72-year-old male with presenting with persistent right-sided facial palsy and arm weakness (NIHSS 3). Admission was chosen by 68% of respondents, with 77% willing to consider randomisation. The factors most frequently reported as influencing the decision to admit included symptom duration (78%), symptom improvement (75%), and social circumstances (74%). Blood pressure (52%), chronic CT abnormalities (53%), and patient age (34%) were also mentioned. Free-text comments highlighted shared decision-making and practical considerations such as bed availability and feasibility of home care.

### Case 2

In this vignette, a 63-year-old female presented with left-sided hypoaesthesia (NIHSS 1). Half of the respondents (50%) opted for admission, while 82% were willing to randomise. Key influencing factors reported were short symptom duration (75%), symptom improvement (64%), and elevated blood pressure (61%). Imaging abnormalities (45%) and social circumstances (42%) were less frequently cited; age was least influential (27%). Several respondents noted patient preference, family context, and considerations about outpatient cardiac monitoring in their free-text responses.

### Case 3

An 85-year-old female with mostly resolved left facial droop and dysarthria (NIHSS 1) was admitted by 60% of respondents, and 94% were willing to randomise. Symptom improvement (83%) and duration since onset (69%) were the dominant reported influences, followed by social circumstances (61%). Age (40%), blood pressure (52%), and CT abnormalities (47%) were less common. Free-text remarks emphasized the influence of advanced age and atrial fibrillation in heightening admission caution.

### Case 4

A 65-year-old male with transient left-sided weakness resolving within 15 min (NIHSS 0) had the lowest admission rate, with only 21% choosing admission and 79% expressing willingness to randomise. Decisions were mostly influenced by symptom improvement (85%) and duration (71%). Approximately half cited CT/CTA abnormalities (56%) and blood pressure (54%). Social circumstances (37%) and age (22%) were less influential. Comments addressed the importance of carotid stenosis evaluation and interdisciplinary collaboration.

### Case 5

This vignette involved a 70-year-old female who presented with aphasia and right facial droop but no residual neurological deficit (NIHSS 0). Admission was favored by 89% of respondents, with 85% willing to randomise. The presence of neuroimaging abnormalities was the strongest factor for admission, reported by 90% of respondents. Duration of symptoms (58%) and clinical improvement (56%) also influenced decisions. Blood pressure (44%) and social circumstances (30%) played lesser roles, while age was cited least (17%). Aphasia, even when resolved, was frequently cited in free-text comments as justification for admission.

## Additional free-text observations

In addition to the predefined factors and the case-specific remarks described above, several recurring themes emerged from the free-text responses. Respondents frequently mentioned the suspected stroke mechanism (e.g., lacunar versus embolic), the potential added value of admission for cardiac monitoring (particularly in patients with atrial fibrillation) and practical or logistical considerations such as bed availability and the role of shared decision-making.

### Acceptable risk of secondary deterioration

When determining acceptable risk levels for secondary deterioration in minor ischaemic stroke, respondents reported that their risk tolerance was modulated by several patient and stroke characteristics. Key factors included the severity of the initial neurological deficit (as measured by NIHSS), patient age, comorbid conditions, and neuroimaging results. Additionally, the presence of robust social support systems and the ability to ensure prompt outpatient follow-up were considered crucial. Within this context, a large majority (89%) of respondents considered a risk threshold of up to 5% for secondary deterioration acceptable to justify direct discharge from the ED. Specifically, 44% of respondents deemed a 1–2% risk of secondary deterioration acceptable, and 45% accepted a 3–5% risk. A notable minority (10%) believed that all ischaemic strokes, even those with minimal neurological deficit, should be admitted regardless of risk level.

## Discussion

This national survey provides a representative overview of the management of minor ischemic stroke in the Netherlands, as reported by 107 Dutch neurologists and neurology trainees. Our findings reveal substantial heterogeneity in admission practices, with rates ranging from 21% to 89% across the clinical vignettes. Despite clear Dutch and international guidelines advocating hospitalization for all acute ischemic stroke cases, these results demonstrate a lack of consensus and indicate that clinicians often adapt their decisions to the perceived short-term risk of deterioration [3, 5, 6]. Cases with minimal or rapidly resolving deficits were less frequently admitted, suggesting that many neurologists (and trainees) already apply an informal risk stratification approach rather than adhering strictly to guideline-driven universal admission. This discrepancy underscores a persistent gap between guideline recommendations and real-world clinical decision-making.

Consistent with prior studies, symptom improvement and duration of deficits were the most decisive factors, influencing 64–85% and 58–78% of respondents, respectively [7, 8]. Social circumstances also weighed heavily (up to 74%), emphasizing the importance of support systems for safe discharge. By contrast, the influence of blood pressure, imaging findings, and age was more variable. Together, these findings confirm that admission decisions for minor stroke extend beyond neurological severity to encompass broader clinical and social determinants. Our data suggest that many neurologists and trainees are already prepared to deviate from guideline-driven routine admission for carefully selected low-risk patients, provided that safeguards are in place. Specifically, 89% of respondents accepted a secondary deterioration risk threshold of up to 5% to justify direct ED discharge, with nearly equal preference for thresholds between 1 and 2% and 3–5%. This pragmatic risk tolerance contrasts with protocols mandating admission for all ischemic stroke cases, reflecting evolving clinical perspectives that balance hospital-related risks, patient preferences, and resource allocation [9]. It should also be recognized that immediate intervention after early neurological deterioration can be challenging regardless of inpatient or outpatient status, as recent literature indicates that delays occur in both settings [7, 10]. 

The observed heterogeneity in decision-making and risk acceptance has important implications for stroke care pathways and future guidelines. More nuanced, evidence-based recommendations are needed that integrate individual risk profiles, clinical features, and social determinants [7, 8, 11]. Developing and validating standardized risk stratification tools could support the identification of patients suitable for safe direct discharge, thereby improving efficiency without compromising safety [8]. Structured care pathways should therefore include comprehensive ED assessment, timely neuroimaging, initiation of secondary prevention, and clear discharge instructions, followed by rapid outpatient follow-up [7, 8]. Equally, patient and caregiver education regarding warning signs and access to emergency services is crucial to mitigate risks associated with early discharge. Recent literature further emphasizes the importance of post-discharge monitoring, even in patients with mild-to-moderate ischemic stroke. Treatable complications such as infections, delirium, and depression can significantly worsen outcomes, underscoring the need for vigilance in all patients regardless of initial stroke severity [12]. At the same time, it is important to recognize that only a minority of patients with neurological deterioration ultimately require acute interventional management, such as thrombolysis, endovascular therapy, or decompressive surgery [7, 8, 10, 11]. 

To our knowledge, this is the first study to systematically examine Dutch neurologists’ and trainees’ attitudes toward admission versus direct ED discharge in minor ischemic stroke. Strengths include the inclusion of both specialists and trainees and the use of clinically realistic vignettes developed by multidisciplinary experts. The high willingness to randomize patients indicates genuine clinical equipoise and provides a strong rationale for conducting future trials. Nevertheless, several limitations must be considered. Hypothetical scenarios, while allowing standardized comparisons, may not fully capture the complexity of real-world decision-making, and participant experience or vignette phrasing could have influenced responses. Although the response rate was sufficient for meaningful analysis, selection bias remains possible as non-responders may hold different views or practices. Reliance on self-reported attitudes and intended behaviors may not always reflect actual clinical practice. Furthermore, while the ABCD2 score is a recognized tool for transient ischemic attack (TIA) risk stratification, it was not directly assessed [13]. Although elements of the ABCD2 score were embedded in our cases, we did not present scores explicitly. Additionally, the absence of formal pilot testing and differences in healthcare system organization limit the generalizability of our findings.

In conclusion, although current Dutch guidelines recommend hospitalization for nearly all patients with minor ischemic stroke, our findings demonstrate considerable variability in clinical practice and an increasing acceptance of selective ED discharge for carefully chosen low-risk individuals. The majority of neurologists and trainees support a tolerable risk threshold of up to 5% for secondary neurological deterioration when considering discharge. These insights highlight the need for evidence-based admission criteria and prospective clinical trials to optimize patient safety and healthcare resource utilization.

## Appendix 1: Overview questionnaire

**Table:** 

Part 1: Respondent characteristics	Age −20-29years −30-39years −30-49years −50-59years −60-70years
	Sex -Male -Female
	Function -Neurologist -Trainnee
	Years since qualifying as a specialist −0-5years −6-10years −11-20years −21-30years ->30years
	Practice -Academic hospital - Major teaching hospital -General hospital -Specialist hospital
**Part 2**: **Treatment questions**	*Would you admit this patient?* -Yes -No
	*Would you be willing to leave this decision (whether to admit or not) open for randomization in a study?* -Yes -No
**Part 3**: **Factors influencing treatment decisions**	*Which of the following factors influence the decision to admit/not to admit?* - Depending on symptom progression - Depending on whether home cardiac monitoring is possible - Insufficient information - Depending on the level of functioning with right arm weakness, can he manage independently
**Part 4**: **Bonus question**	Regarding stroke with minor neurological deficit for which no acute treatment is given, what percentage of risk for secondary deterioration would you accept for direct ED discharge? - 1–2% - 3–5% - 6–10% - Everyone with a stroke with minor neurological deficit should be admitted

## Data Availability

No datasets were generated or analysed during the current study.
